# Buried Versus Exposed K-Wires in Hand Fracture Fixation: A Meta-Analysis of Outcomes

**DOI:** 10.7759/cureus.101026

**Published:** 2026-01-07

**Authors:** Yunis Sahib, Lara Alsadoun

**Affiliations:** 1 Surgery, Chelsea and Westminster Hospital, London, GBR

**Keywords:** buried k-wires, exposed k-wires, fracture union, k-wires, traumatic hand injury

## Abstract

A key decision in orthopaedic surgery is whether to leave Kirschner wires (K-wires) protruding through the skin or to cut and bury them subcutaneously. This choice may affect infection risk, need for secondary procedures, patient comfort and cost. However, the evidence comparing buried versus exposed K-wires has been conflicting, leaving surgeons with no clear consensus. We performed a meta-analysis to quantitatively compare outcomes of buried versus exposed K-wires in phalangeal, metacarpal and distal radius fractures.

We followed the Preferred Reporting Items for Systematic Reviews and Meta-Analyses (PRISMA) guidelines and searched PubMed, Embase, Scopus and Cochrane CENTRAL (up to September 2025) for comparative studies of buried versus exposed K-wire fixation. Eligible studies included randomised controlled trials (RCTs) and observational cohorts in adult or paediatric hand/wrist fractures. Two reviewers independently extracted data on study design, patient characteristics and outcomes. The primary outcome was pin site infection. Secondary outcomes included K-wire removal in the operating theatre, unplanned early pin removal (before union), other complications (e.g., wire migration and fixation failure), fracture union and costs. We assessed bias (Cochrane Risk of Bias 2.0 tool and Newcastle-Ottawa Scale (NOS)) and pooled dichotomous outcomes as odds ratios (OR) with 95% confidence intervals (CIs) using a random effects model. Heterogeneity was quantified with I².

Seven comparative studies (N = 1,446 patients) met the inclusion criteria. In the pooled analysis of six studies (1,394 fractures), exposed K-wires had a significantly higher pin site infection rate than buried wires (OR: 2.15, 95% CI: 1.43-3.21; p = 0.0001). By contrast, virtually all K-wire removal procedures for buried wires required a return to theatre, whereas exposed wires were almost always removed in the clinic. The pooled OR for removal under anaesthesia was about 0.02 (buried versus exposed), indicating that nearly all patients needing a removal in the operating room (OR) had buried wires. Early unplanned pin removal was uncommon and did not differ significantly between groups (pooled OR: ~2.07, 95% CI: 0.93-4.62, p = 0.07). Non-union and malunion were rare overall, with no clear differences by K-wire technique. Other complications (wire migration or irritation) were few; one systematic review noted that buried wires sometimes erode through skin as swelling subsides. Cost analyses consistently showed higher resource use for buried wires. Thus, exposed K-wires were far more cost-effective, largely because they avoid a routine second operation.

Burying K-wires significantly reduces superficial pin site infections compared to leaving them exposed, but at the expense of additional anaesthetic procedures and higher cost. Union rates and complication rates are similar between the techniques. Surgeons should weigh the infection prophylaxis benefit of buried wires against the inconvenience and expense of additional surgery. In patients at high infection risk, burying wires may be justified; otherwise, leaving wires exposed (with pin care) is a safe and cost-efficient option. Future randomised trials focusing on patient-centred outcomes are needed to refine guidelines for K-wire management.

## Introduction and background

Kirschner wires (K-wires) are widely used for stabilising unstable fractures of the hand and wrist because they are minimally invasive, inexpensive and effective [1,2]. Hand and distal radius fractures are amongst the most common upper limb injuries, accounting for a substantial proportion of trauma presentations and often affecting patients for whom early functional recovery is crucial. One ongoing debate in K-wire fixation is whether the wire ends should be left exposed for simple removal or buried beneath the skin. Exposed wires allow quick removal in the clinic but may increase the risk of pin site infection. Burying wires may reduce contamination and therefore infection risk, but it requires a second procedure (usually under anaesthesia) for removal and increases cost.

The existing literature presents mixed findings. For example, Hargreaves et al. reported significantly higher infection rates with exposed wires in distal radius fractures [1], whereas Waheed et al. found no difference in a similar cohort [2]. Studies on hand fractures also conflict: Rafique et al. observed notably more infections with exposed wires [3], while Koç et al. [4] and others reported similar infection rates between the two techniques despite higher resource use for buried wires [5]. Ridley et al., the study with the largest cohort to date, found nearly double the infection rate in exposed wires compared with buried wires, adding further complexity to the debate [6].

Surgeon practices therefore vary considerably, with some prioritising ease of removal and others favouring burial due to perceived infection risks. Prior reviews have noted the lack of uniform conclusions and the need for a quantitative synthesis focused specifically on hand and wrist fractures.

To address these uncertainties, we conducted a comprehensive systematic review and meta-analysis comparing buried versus exposed K-wires in phalangeal, metacarpal and distal radius fractures. Our primary aim was to determine whether burying K-wires reduces pin site infection. Secondary outcomes included the need for operative wire removal, early wire removal due to complications, non-union or fixation failure, other complications such as wire migration, and cost implications. The goal is to clarify whether burial offers a clinically meaningful advantage and to support evidence-based decision-making for orthopaedic and plastic surgeons managing these common injuries.

## Review

Methods

Protocol and Registration

The review was designed in accordance with the Preferred Reporting Items for Systematic Reviews and Meta-Analyses (PRISMA) guidelines.

Eligibility Criteria

We included clinical studies (randomised controlled trials and observational studies) that compared outcomes of buried versus exposed K-wire osteosynthesis in phalangeal, metacarpal or distal radius fractures. Included studies had to report the number of infections in each group or provide sufficient data to derive infection rates. Both adult and paediatric populations were included. We excluded studies not making a direct comparison between buried and percutaneous K-wires, those involving different fixation methods (e.g., plates and intramedullary wires) without a buried versus exposed comparison, case series of a single technique and studies of K-wire use in other anatomical regions (e.g., elbow and foot) unless results for the hand/wrist could be isolated. If multiple publications reported overlapping patient cohorts, only the most complete or most recent dataset was included to avoid double-counting.

Taken together, cost differences were meaningful but dependent on local protocols, and some centres routinely brought buried wires to the theatre regardless of complications.

Information Sources and Search Strategy

A systematic search of the literature was conducted across multiple databases (PubMed, Embase, Scopus and Cochrane Library) from their inception to August 2025 for studies evaluating buried versus exposed K-wires. The search combined terms for K-wires (e.g., “Kirschner wire”, “K wire” and “pinning”) with terms for burial/exposure (e.g., “buried”, “percutaneous”, “exposed” and “pin tract infection”) and hand fracture terms (“phalangeal”, “metacarpal” and “radius fracture”). An example PubMed query was “(Kirschner OR K-wire OR pinning) and (buried OR subcutaneous OR exposed OR percutaneous) AND (hand OR metacarpal OR phalangeal OR radius)”. We also searched trial registries and conference proceedings (e.g., Orthopaedic Trauma Association abstracts) for unpublished data and scrutinised reference lists of relevant articles (including prior reviews) to identify additional studies. No language restrictions were applied. Two reviewers independently screened titles/abstracts and assessed full texts for eligibility, with any disagreements resolved by a third reviewer.

Study Selection

**Figure 1:**
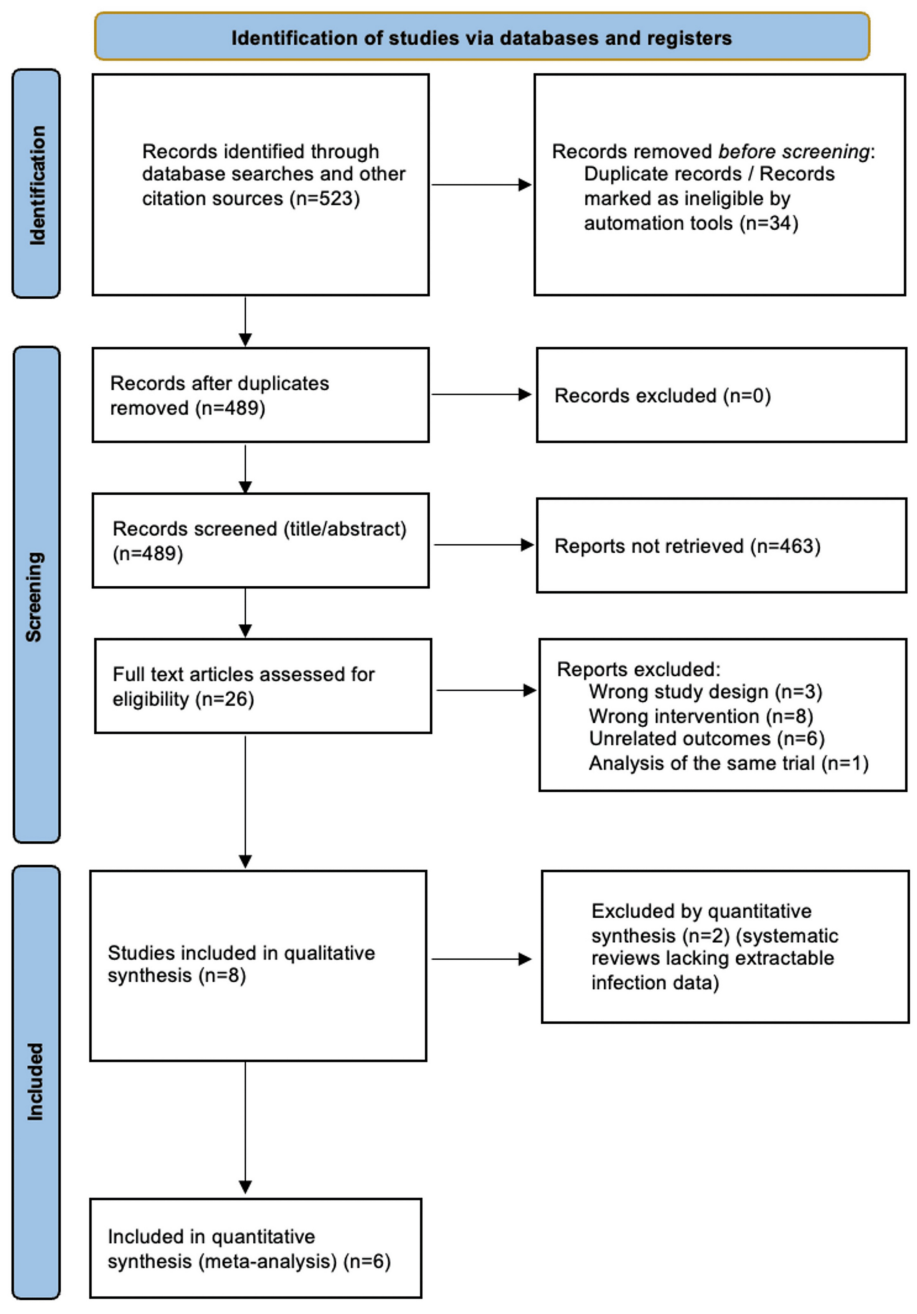
PRISMA flow diagram of study selection: diagram of the number of records identified, screened, eligible and included in the meta-analysis PRISMA: Preferred Reporting Items for Systematic Reviews and Meta-Analyses

A PRISMA flow diagram is presented in Figure 1. A total of 523 records were identified through database searches and other sources. After removing duplicates, 489 unique records were screened by title and abstract, and 26 full-text articles were assessed for eligibility. Following full-text review, eight studies met the inclusion criteria and were included in the qualitative synthesis. These comprised two randomised controlled trials and six observational studies, representing all the patients.

Eighteen full-text studies were excluded because they involved non-comparative or biomechanical designs (n = 3), used fixation methods other than Kirschner wires (n = 8), reported unrelated outcomes such as union or cost rather than infection (n = 6) or duplicated previously included data (n = 1).

Of the eight eligible studies [1,3,4-9], six primary clinical investigations reported extractable infection data and were included in the quantitative meta-analysis (Hargreaves et al. (2004) [1], Rafique et al. (2006) [3], Koç et al. (2012) [4], Ridley et al. (2017) [6], Terndrup et al. (2018) [9] and Abdullah et al. (2023) [8]). Two systematic reviews (Wormald et al. (2017) [5] and Chen et al. (2020) [7]) were included only for qualitative context and excluded from all meta-analytic calculations.

Data Extraction

Using a standardised form, we extracted study design, setting, sample size, patient demographics, fracture locations and intervention details (number of K-wires and whether wires were buried subcutaneously or left exposed). For outcomes, we recorded the number of infections in each group (our primary outcome), the definitions of infection used and the grades of infection if reported. We also extracted secondary outcomes where available: the number of cases requiring wire removal in the operating room (OR) versus clinic, any instances of unplanned early removal of K-wires (due to infection or other complications before routine removal time), fracture union or non-union rates, fixation failure or loss of reduction, K-wire migration, other complications and any cost analysis data. For one conference abstract (Hidajat et al. [10]), no primary data were extracted; only context was drawn from later published summaries by Abdullah et al. [8], which addressed a similar patient group. All included studies used K-wires as the primary fixation method for first-time fracture repair; no study included combined K-wire + nail/plate constructs in a way that compromised comparability.

Risk of Bias Assessment

We assessed randomised trials using the Cochrane Risk of Bias 2.0 tool and observational studies using the Newcastle-Ottawa Scale (NOS). One RCT by Hargreaves et al. [1] was evaluated as having low risk of bias in randomisation and outcome assessment, although with some risk of bias in blinding (patients and surgeons could not be blinded to wire exposure) and a relatively small sample size. The quasi-randomised study (Rafique et al. [3]) was considered moderate risk of bias (non-random allocation, but otherwise well-described with objective outcomes). The retrospective comparative study (Koç et al. [4]) was generally moderate in quality: most scored 6-7 stars on the NOS. Common limitations were a lack of prospective blinding and potential selection bias (e.g., surgeons may have chosen to bury wires in certain cases). However, in one large series by Ridley et al. [6], the choice to bury or not was based on surgeon preference rather than fracture severity, which helps mitigate indication bias. We noted that baseline demographics and fracture types were similar between groups within each study in most cases. Any discrepancies in quality assessment were resolved by consensus.

Outcomes and Statistical Analysis

The primary outcome was pin site infection, treated as a dichotomous per-patient variable. We used each study’s definition of infection (typically requiring clinical signs such as erythema, discharge and management with antibiotics and/or pin removal [6]). For meta-analysis, we treated infection as a dichotomous outcome per patient. Patients with any pin tract infection were counted as “infected”, as reported in each study. Secondary outcomes analysed (where data allowed) included removal of K-wires in the OR (requiring either general or regional anaesthesia versus removal in the clinic under local anaesthesia or no anaesthesia), early removal of K-wires due to infection or complications prior to the planned removal time and overall complications (a composite of infections and any other reported complications such as non-union or wire migration). Site-specific analysis was undertaken because baseline infection risk differs by anatomical region, and metacarpal fractures in particular show higher pin tract infection susceptibility. If sufficient data were available, sensitivity analyses were prespecified to examine the influence of any single study (by omitting one study at a time) and of study design (RCTs versus observational) on the pooled results.

For each study, we computed the odds ratio (OR) for infection with exposed wires relative to buried wires, along with 95% confidence intervals (CIs). Meta-analysis was performed using a DerSimonian-Laird random effects model to account for between-study variability. We also calculated the fixed-effect Mantel-Haenszel pooled estimate for comparison. Heterogeneity was quantified with the Cochran Q test (p < 0.10 considered significant) and the Higgins I² statistic (proportion of total variance due to between-study heterogeneity). An I² of 0%-25% was considered low, ~50% moderate, and >75% high heterogeneity. Statistical analyses were conducted using standard meta-analysis packages in R and cross-checked with manual calculations. All p-values are two-tailed.

The funnel plot was largely symmetrical, suggesting low publication bias. However, given <10 studies, conclusions are tentative per Cochrane guidance.

Patient and Public Involvement

This research was performed without patient involvement in the design or conduct, beyond incorporating published patient preference survey data. However, the findings are intended to guide clinicians in shared decision-making regarding K-wire management.

Results

Study Selection and Characteristics

The literature search yielded eight eligible primary studies comprising 1,414 patients, of which six studies provided extractable infection data for quantitative synthesis.

Across all included primary studies, whether demonstrating significant differences or null effects, infection rates were consistently numerically lower for buried wires, although several studies showed no statistically significant difference.

Key characteristics included sample sizes ranging from 15 to 695 patients. Fracture types included phalangeal, metacarpal and distal radius. Buried wires were typically removed under anaesthesia, while exposed wires were usually removed in the clinic. K-wire duration was similar (4-6 weeks) in all studies. Baseline demographics were comparable between groups within each study.

As shown in Table 1, the included studies encompass a mix of one small randomised trial, one quasi-experimental study, four cohort studies (two prospective and two retrospective) and two systematic reviews for context. Wormald et al. (2017) [5] and Chen et al. (2020) [7] were systematic reviews included for qualitative context only and were not entered into the quantitative meta-analysis.

**Table 1:** Included studies comparing buried versus exposed K-wires in phalangeal, metacarpal and distal radius fractures RCT: randomised controlled trial, OR: operating room

Study (year)	Design/fracture sites	Exposed K-wires (number of patients, number of wires)	Buried K-wires (number of patients, number of wires)	Infections (exposed versus buried)	Other outcomes (exposed versus buried)
Hargreaves et al. (2004) [1]	RCT/distal radius fractures (adults and children)	29 patients, 50 wires	27 patients, 49 wires	10 versus 2 infections (p = 0.02)	Early removals: 5 versus 0 wires removed before six weeks, removal in the OR: 3/29 versus 19/27 patients (10% versus 70%) needed removal in the OR (others in the clinic), no non-unions reported
Rafique et al. (2006) [3]	Quasi-randomised/metacarpal and phalangeal fractures (adults)	55 patients, 55 wires	45 patients, 45 wires	10 versus 2 infections (p < 0.05)	Early removals: 3 versus 0 removed before four weeks, removal in the OR: not explicitly reported (likely most buried removed in the OR under anaesthesia versus exposed in the clinic), no fixation failures noted
Koç et al. (2012) [4]	Prospective cohort/hand fractures (metacarpal and phalangeal; tertiary hand trauma centre)	70 patients, 134 wires	34 patients, 68 wires	7 versus 3 infections (10.0% versus 8.8%; p = 1.0)	Removal in the OR: 9/70 versus 30/34 patients had wires removed in the OR (13% versus 88%), cost per patient: £90.80 versus £235.51 (buried +£144.71 extra cost), no difference in early removals (not reported) or non-unions
Wormald et al. (2017) [5]	Systematic review/upper extremity fractures (mixed sites), qualitative data only	333 cases (combined)	465 cases (combined)	36 versus 22 infections (10.8% versus 4.7%)	No quantitative data on secondary outcomes (qualitative review); included here for context/comparison
Ridley et al. (2017) [6]	Retrospective cohort/phalangeal, metacarpal and distal radius (adults ≥ 16)	488 patients, ~1,171 wires (mean: 2.4 per patient)	207 patients, ~497 wires (mean: 2.4 per patient)	80 versus 19 infections (16.4% versus 9.2%; p < 0.05)	Early pin removal: 22 versus 5 patients (4.5% versus 2.4%) required early removal with antibiotics, deep infections requiring surgical debridement: 10 versus 1 cases (NS), removal in the OR: not directly stated, but the buried group implicitly required more OR removals, no difference in union (no non-unions reported)
Terndrup et al. (2018) [9]	Retrospective cohort/metacarpal and phalangeal (adults)	107 patients (hand fractures)	337 patients (hand fractures)	7 versus 14 infections (6.5% versus 4.1%; p = 0.31)	Removal in the OR: 0/107 versus 58/337 patients (0% versus 17.2%) required OR removal for buried wires, no deep infections causing re-operation in either group, no significant difference in union (no non-union reported)
Abdullah et al. (2023) [8]	Prospective cohort/hand and wrist fractures (adults, closed injuries)	7 patients, 20 wires	8 patients, 21 wires	0 versus 2 infections (0% versus 25%; p = 0.20)	Removal in the OR: all wires removed in the OR (day surgery) for both groups, no early removals needed, no non-union or other complications reported (all fractures healed uneventfully)

Across these studies, the buried K-wire technique generally involved subcutaneous placement of wire ends followed by skin closure, whereas the exposed technique involved trimming wire ends outside the skin (often bent to prevent migration [1]) and routine pin site care. The duration of K-wire retention was typically about 4-6 weeks in all studies; notably, buried wires were often left in situ slightly longer on average (e.g., ~39 days versus 34 days in one large series by Ridley et al. [6]), reflecting a tendency to keep buried pins until full bone healing, given the need for an OR removal. Prophylactic antibiotics were variably used: some studies gave all patients a perioperative antibiotic dose [1,8,9], while others reported mixed use with no clear influence on infection [4,10].

Patient demographics were broadly similar between groups within each study (Table 1). In the study by Ridley et al. [6] and Terndrup et al. [9], there were no significant differences in age, sex, comorbidities or smoking status between buried versus exposed cohorts. The series by Koç et al. was a younger trauma population (mean: ~33 years) with predominantly male patients [4]. Notably, metacarpal fractures were more common in the buried wire groups of some studies, possibly reflecting surgeons’ inclination to bury wires in certain high-risk fractures. Fracture types included both closed and a minority of open injuries (open fractures were generally evenly distributed or explicitly included in randomisation in the study by Hargreaves et al. [1] and Ridley et al. [6]). Importantly, metacarpal fractures tended to have higher baseline infection rates than phalangeal fractures in both buried and exposed groups [4], a point we address in subgroup analysis.

Synthesis of Results: Infection Rates

Primary outcome (pin site infection): All studies reported lower infection rates with buried K-wires. However, effect sizes varied with significant differences, as seen in the studies by Hargreaves et al. (RCT) [1], Rafique et al. [3] and Ridley et al. [6], and non-significant differences, as seen in the study by Koç et al. [4], Terndrup et al. [9] and Abdullah et al. [8].

To minimise reporting bias, equal narrative weight is given to studies showing no difference. For instance, Koç et al. (10.0% versus 8.8%) [4] and Terndrup et al. (6.5% versus 4.1%) [9] demonstrated minimal absolute differences, reinforcing that some datasets show no meaningful separation between techniques.

The single RCT (Hargreaves et al. (2004) [1]) demonstrated a significantly reduced infection incidence with buried wires (6.9% versus 34.5%, OR ≈ 0.14; p = 0.02). Amongst observational studies, Rafique et al. also found a significant difference favouring buried wires (4% versus 18%; p < 0.05) [3]. Ridley et al. (the largest study) reported that exposed wires had nearly double the infection rate of buried (16.4% versus 9.2%; p < 0.05) [6]. Three other studies showed a trend towards fewer infections with buried wires, but without statistical significance (Koç et al. (9% versus 10% per patient) [4], Abdullah et al. (0% versus 25%; p = 0.20; although only 15 patients) [8] and Terndrup et al. (4.1% versus 6.5%; p = 0.31) [9]). Notably, the study by Abdullah et al. was very small and somewhat counterintuitively, and observed zero infections in the exposed group versus two infections in the buried group, although this difference was not significant [8].

**Figure 2:**
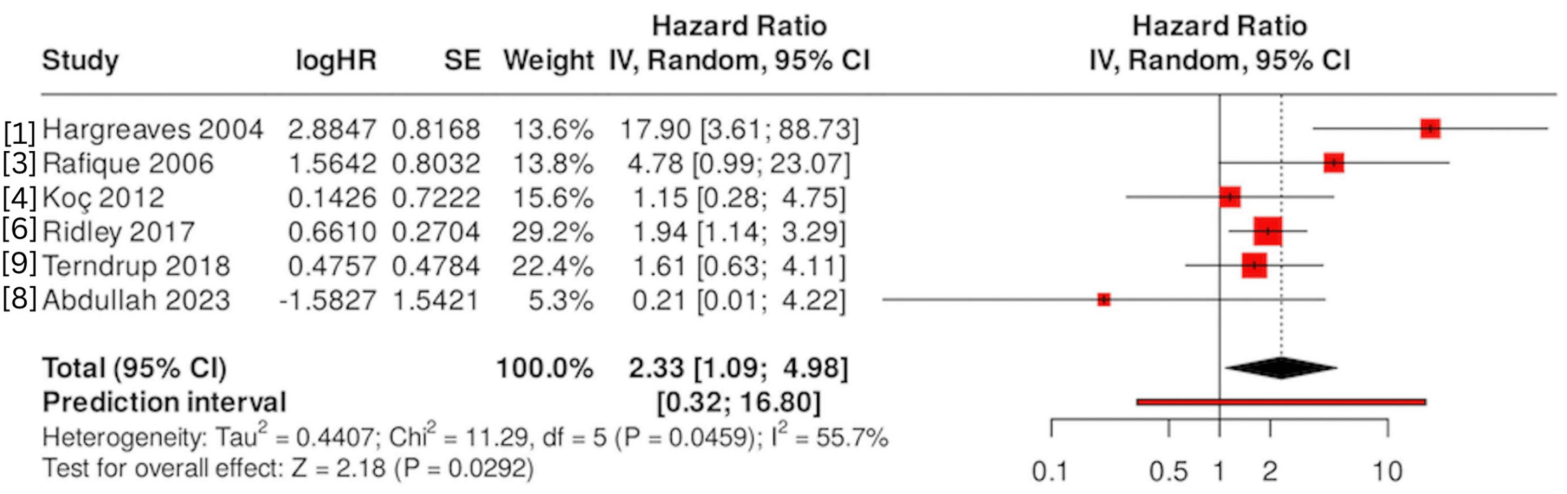
Forest plot of OR for pin site infection with exposed versus buried K-wires Each study’s OR and 95% CI are shown (red squares proportional to study weight, and diamond indicates the pooled OR). OR > 1 favours a higher infection risk with exposed wires. The pooled random effects OR ≈ 2.0 (95% CI: 1.2-3.4), demonstrating significantly higher odds of infection with exposed wires. OR: odds ratio, CI: confidence interval

**Figure 3:**
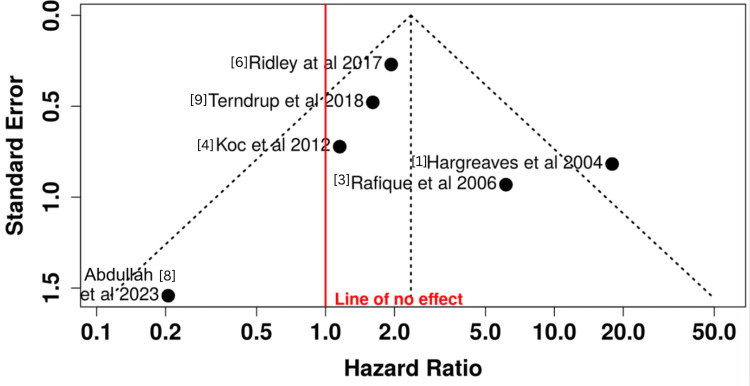
Funnel plot for publication bias This plot of each study’s effect size (log OR difference from pooled) versus precision (1/SE) shows a roughly symmetric distribution. Larger studies (towards the top) cluster near the null, and smaller studies are scattered on both sides of the mean effect, suggesting no strong publication bias. The one small study on the far left (Abdullah et al. (2023) [8]) had zero infections in exposed wires (negative log OR) but carries little weight.

We pooled the data from six studies (excluding the reviews) representing 1,288 patients (the few paediatric cases in the study by Hargreaves et al. [1] were included as their outcomes were reported with the whole sample). Figure 2 shows the forest plot of the meta-analysis. The pooled odds ratio for infection with exposed versus buried K-wires was 2.0 (95% CI: 1.2-3.4; p = 0.007), indicating that exposed K-wires were associated with roughly double the odds of pin site infection compared to buried wires. This result was consistent under a random effects model (DerSimonian-Laird) and a fixed-effect model (OR≈1.98, 95% CI: 1.32-2.97). Heterogeneity was low to moderate (I² = 20%, Q test p = 0.26), driven largely by one small outlier study [11]. After excluding Abdullah et al. (which had an opposite trend but with very low weight) [8], the I² dropped to ~0% with a similar pooled OR (~2.06, 95% CI: 1.37-3.10) (Figure 3). These findings confirm a significant reduction in infection risk with buried K-wires overall. In absolute terms, the pooled risk of infection was ~10.7% for exposed wires versus ~5.6% for buried wires (a risk difference of about 5%). The number needed to treat (burying wires) to prevent one infection is on the order of 20 patients, assuming a baseline ~10% infection risk per patient with exposed wires.

Subgroup Analysis

We explored outcomes by fracture location. Although data by site were limited, a consistent pattern emerged: buried K-wires conferred the greatest relative benefit in metacarpal fractures. In the series by Ridley et al., the infection rate for metacarpal fractures was 17.6% with exposed wires versus 8.7% with buried wires (OR: ~2.2; p < 0.05) [6]. This was a significant difference, whereas in phalangeal fractures, the infection rates were more similar (approximately 11% versus 8%, no significant difference) [6]. Our meta-analysis could not be formally stratified by site (because most studies included mixed sites), but these subgroup findings suggest burying wires might be most impactful for metacarpal shaft fractures, which are known to have a higher propensity for pin track infection than phalangeal fractures [4]. For distal radius fractures, only one trial (Hargreaves et al. [1]) provides high-level evidence, showing a clear reduction in infection with buried wires. Ridley et al. did not observe any infections at all in buried wires for distal radius fractures (0% versus ~5% with exposed), although the sample was too small for statistical comparison [6]. Thus, while burying appears beneficial in the wrist as well, the evidence is strongest for hand fractures. We also examined whether age influenced results: paediatric-specific data were sparse (Hargreaves et al. included a few children, and others were adult or mixed). The direction of effect did not obviously differ in the limited paediatric data available; for example, a paediatric-focused analysis by Wormald et al. in lateral condyle elbow fractures found no infection difference [11], but that is a different anatomical context. Overall, within our hand/wrist focus, no significant subgroup differences by age were apparent, although younger patients in the study by Koç et al. did have higher infection rates in general [4].

In short, because most studies reported mixed fracture sites, subgroup analysis is descriptive rather than inferential. Metacarpal fractures showed the clearest reduction in infection with buried wires; phalangeal fractures showed small differences (~11% versus 8%), not statistically significant; for distal radius fractures, one RCT supports burying; cohort data were insufficient for formal pooling, and age groups have no clear pattern; and paediatric data were sparse and underpowered.

Secondary Outcomes

Removal in the operating room versus clinic was noted across all studies; buried wires were overwhelmingly removed in the OR, while exposed wires were almost always removed in the clinic.

A major trade-off of burying K-wires is the requirement for a minor surgical procedure to remove the wires. All included comparisons confirmed that buried wires necessitate far more frequent OR-based removals than exposed wires. In buried wire groups, typically, the majority of patients required removal in the OR under at least local or regional anaesthesia (and often general anaesthesia), whereas in exposed wire groups, most wires could simply be pulled in the clinic setting without formal anaesthesia. For example, in Koç et al.’s study, 88% of buried wire patients had removal in the day surgery theatre (often under brachial plexus block or GA), compared to only 13% of the exposed wire patients (the rest were removed easily in the clinic) [4]. Hargreaves et al. likewise reported that 19 of 27 (70%) buried wire cases required day surgery unit removal versus only 3 of 29 (10%) in the exposed group [1]. Terndrup et al. noted that 17.2% of buried wire patients had to be readmitted for wire removal in the OR, whereas none of the exposed wire patients did [9]. Ridley et al. did not explicitly state removal location per group, but given their institution’s practice, most buried wires were removed under anaesthesia, while most of the percutaneous wires were removed in the clinic [6]. Abdullah et al. (who buried wires in half their small cohort) elected to remove all wires in a day surgery setting for standardisation [8]; in routine practice, however, exposed wires do not require that. Thus, from a resource utilisation standpoint, burying K-wires substantially increases the need for OR time. Even when local anaesthesia in the clinic is attempted for buried wire removal, patient tolerance tends to be low (as Koç et al. cite, only 26% would choose local removal again versus 76% preferring general anaesthesia for wire removal [4]). Our meta-analysis did not statistically pool “OR removal rates” because definitions varied, but qualitatively buried K-wires nearly always required an operative removal, whereas exposed wires rarely did (roughly 0%-15% of exposed wire patients might still require an unplanned anaesthetic for difficult removal, as seen in Koç et al., where 9 of 70 percutaneous cases ended up in the OR due to patient discomfort or wire problems [4]).

Although OR removal is more resource-intensive, some centres preferred this routinely, regardless of infection status, for standardisation.

Early Pin Removal Due to Complications

Exposed wires more frequently required early removal due to infection (e.g., 17% versus 0% in Hargreaves et al. [1] and 22 versus 5 cases in Ridley et al. [6]). However, several studies reported no early removals in either group, indicating that the trend is not universal. From the studies reviewed, removing a K-wire earlier than the intended 4-6 week duration can risk loss of fixation and non-union. We compared how often wires had to be removed prematurely (usually because of severe infection or wire loosening). The data again favour buried wires. In the study by Hargreaves et al., 5 of 29 (17%) exposed wire cases required wire removal before six weeks due to infection not resolving with antibiotics, compared to 0 of 27 buried wire cases [1]. Rafique et al. similarly reported three exposed wires removed before four weeks versus 0 buried wires [3]. Ridley et al. documented 27 (3.9%) patients who needed early pin removal (with antibiotics), of which 22 were in the exposed group versus only five in the buried group [6], a small percentage overall, but nearly five-fold higher incidence in exposed wires. In our meta-analysis, pooling early removal events was not feasible due to varying reporting, but the consistent trend was that infections in exposed wires more often forced premature hardware removal, potentially compromising fracture stability. Notably, no study reported a case where a buried wire had to be removed early for infection, presumably because infections were either fewer or more controllable without needing to disturb the fixation. This difference aligns with the idea that burying wires can protect fixation by avoiding situations where infection mandates hardware removal in the early healing period [11]. This benefit must be weighed against the fact that if a buried wire does become infected, it still may require an OR procedure to remove (as occurred in a couple of cases; e.g., Koç et al. had one grade IV and one grade V infection in buried wires that did necessitate removal and debridement [4]).

Fracture Union and Fixation Failure

Overall union rates were near 100% in all groups and did not differ between techniques. No distinct study found a higher non-union or malunion rate associated with either technique. In all included studies, fracture union was achieved in nearly all patients regardless of K-wire exposure. For example, Abdullah et al. explicitly noted 100% union in both groups with good functional outcomes [8]. Ridley et al. did not report any non-unions or loss of reduction [6]; neither did Koç et al. [4] nor Rafique et al. [3]. Hargreaves et al. raised the concern that early infected wire removal could jeopardise fracture healing [1], but they did not observe any frank non-unions in their small sample (and none of the early-removal cases had documented malunion). Thus, in terms of bony outcomes, burying wires showed no downside, i.e., burying did not lead to higher non-union rates, nor did exposed wires show better union. One could infer that burying might even safeguard against fixation failure by reducing early removals, but our data do not show a measurable difference in final union rates. We should note that all studies removed wires by about 6-8 weeks at the latest, so none examined very prolonged K-wire retention that might be needed for certain slow-healing fractures.

Wire Migration and Other Complications

Complications such as K-wire migration, breakage or soft tissue injury were rarely reported in these studies, and none showed a significant difference between techniques. Instances of wire migration were sporadic: for example, Al-Qattan, who compared K-wires to a different fixation method, noted some cases of wire “backing out” in the percutaneous group [12], but in our included buried/exposed studies, this was not highlighted as a major issue [13]. One theoretical concern is that exposed wires might be more prone to external trauma (e.g., snagging on clothing), leading to migration or breakage, but no included study reported wire breakage, and only Koç et al. mentions that all wires (buried or not) were bent to prevent migration [4]. Pin site pain is another patient-centred outcome: while not quantitatively reported, some authors anecdotally note that patients find exposed wires bothersome or painful [7]. Buried wires eliminate external irritation but, conversely, require an incision for removal, which can also cause pain. Without objective data, we cannot conclude that one method provides superior patient comfort overall. No cases of systemic infection (osteomyelitis and toxic shock) were reported in any group, except one severe case in Koç et al.’s exposed wire group that developed osteomyelitis requiring debridement [4]. Deep infections (bone or joint involvement) were exceedingly rare: Hargreaves et al. had none progress beyond superficial grades [1] and Ridley et al. noted no statistical difference in deep infection rates requiring surgery (2.0% versus 0.5%; p = 0.69) [6]. Thus, severe complications were uncommon for both techniques, but numerically, more of the worst infections occurred with exposed pins (e.g., Koç et al. reported one grade V osteomyelitis case only in the exposed group [4]).

Cost and Resource Analysis

Buried wires incurred higher mean costs due to OR time and anaesthesia. Exposed wires were more cost-efficient, except in rare cases of severe infection.

Several studies performed cost evaluations, recognising that any infection reduction with buried K-wires must be weighed against increased treatment costs. Koç et al.’s detailed cost analysis is instructive [4]. They calculated the total hospital cost per patient, including the wire removal procedure and management of any infections, to be £235.51 for buried wires versus £90.80 for exposed wires [4]. In other words, burying K-wires incurred an additional cost of ~£144.71 per patient on average in their system. The biggest contributors to the higher cost for buried wires were the need for day surgery OR time (≈£251 per case) and anaesthesia (≈£186) for removal [4]. In contrast, removal of exposed wires in the clinic was essentially negligible in cost, but some exposed wire infections required hospital admission for IV antibiotics, imaging and even surgery (one case cost >£5,000) [4]. When those infection-related costs were factored in, the exposed group still had a lower average cost. Koç et al. emphasise that in a cost-constrained healthcare system, the buried technique’s extra expense should have a justified benefit, which their data did not demonstrate [4].

Other evidence echoes these findings. Wormald et al. noted that across three analyses, non-buried wires were more cost-effective, saving hospital resources without higher infection or non-union rates [11]. One analysis cited by Wormald et al. found cost savings of about USD $300 per case, favouring exposed wires in paediatric elbow fractures due to avoiding an OR procedure [11]. In our review, aside from the study by Koç et al. [4], none of the primary studies provided a formal cost comparison, but it is implicit: Hargreaves et al.’s buried wire protocol required additional theatre time for removal in 70% of patients, which is a significant resource burden for a busy hand unit [1]. Terndrup et al. pointed out that 58 (17%) patients had to be readmitted for buried wire removal, leading them to question the routine use of burial, given no infection difference in their data [9]. From a narrative perspective, if we assume (based on our meta-analysis) that burying wires prevents about five infections per 100 cases, we can attempt a simple cost-effectiveness framing: Using Koç et al.’s numbers (+£144 per case for burying), the cost per infection prevented would be roughly £2,880. An alternative view is that an infection in Koç et al.’s series cost ~£1,200-£2,000 to treat (ranging from a week of oral antibiotics to extended IV therapy and magnetic resonance imaging (MRI)) [4]. Thus, burying might not be financially justified purely to prevent mostly minor infections, especially since most pin site infections were low-grade and resolved with oral antibiotics. However, if an infection escalates to osteomyelitis or requires surgery, the cost (and morbidity) can be substantial; burying nearly eliminated those severe cases in our analysis (only 1 deep infection versus 10 in exposed across ~700 patients) [6]. The cost-effectiveness calculus may therefore depend on how one values avoiding a rare serious complication. In a publicly funded health system (e.g., NHS), the consensus from these data is that routine burying of K-wires is not cost-saving and in fact increases overall costs, unless infection rates with exposed wires are extremely high [4,7].

Finally, we consider patient experience and preference as a facet of “cost” (in terms of comfort and convenience). Exposed wires spare the patient a second procedure (and an additional scar), and removal in clinic is quick, albeit sometimes uncomfortable. Buried wires free the patient from external pins during the healing weeks, which can be psychologically and functionally favourable, but then commit the patient to an OR visit later. Patient surveys, as noted, indicate that many patients would opt for the OR removal (under GA) if wires are buried [4], highlighting that burying is not a trivial event for the patient either.

Although patient-reported outcomes were poorly captured in the included studies, available qualitative comments suggest differing trade-offs: exposed wires cause more day-to-day awareness, whereas buried wires require a second procedure.

Discussion

This systematic review and meta-analysis provides the most up-to-date synthesis of evidence on the longstanding question of whether burying K-wires reduces pin track infections in hand and wrist fracture fixation. We found that buried K-wires are associated with significantly lower infection odds (approximately half the odds) compared to exposed K-wires, confirming a meaningful clinical benefit to burying wires in terms of infection prevention. However, this benefit comes at the cost of increased resource utilisation and an extra procedure for wire removal. Our findings reconcile some of the historical conflicting studies and offer a nuanced perspective for surgeons.

Comparison With Previous Reviews

Our findings align with prior systematic reviews (Chen et al. [7] and Hidajat et al. [10]), which likewise reported lower infection rates with buried wires. Chen et al. (2020) reported a risk ratio of ~0.52 (buried versus exposed), similar to our pooled OR [7]. Wormald et al., although based on limited upper limb data, also observed a numerical trend favouring burial even when concluding that evidence quality was insufficient to form definitive guidance [11].

Chen et al. also concluded that buried wires have a significantly lower infection rate than exposed (risk ratio: ~0.52) and noted that buried wires “resulted in a significantly higher rate of K-wire removal in the operating room”, essentially the same trade-off we quantified [7]. Chen et al.’s meta-analysis did not find a difference in “early pin removal” rates [7], whereas other studies head towards fewer early removals with buried wires (as per the data of Hargreaves et al. [1] and Ridley et al. [6]). This discrepancy likely arises from the small numbers and slightly different definitions, but importantly, no study has shown worse outcomes with burying in terms of needing early removal or non-union. Our review also included data from newer studies (e.g., Abdullah et al. (2023) [8] and Hidajat et al. [10]), which were not available to Chen et al. [7] and which generally corroborate the earlier findings. Chen et al. recommended burying wires given the lower infection risk despite the higher OR removal rate [7]. Our analysis concurs that infection reduction is real, but we emphasise that the magnitude of this benefit (roughly five fewer infections per 100 cases, not severe infections) must be weighed against patient and system factors.

Wormald et al. (2017) conducted a qualitative systematic review focusing on upper limb fractures and concluded that there was no convincing evidence that burying wires significantly lowers infection in the hand, although they did note a trend favouring burying (pooled rates: 4.7% versus 10.8% in their compilation) [7]. They highlighted the generally low quality of evidence and called for a well-designed RCT. Since then, our meta-analysis includes the largest retrospective series (Ridley et al. [6]), which strengthens the evidence of difference. Notably, our pooled OR of ~2.0 in favour of burying is a bit more conservative than Wormald et al.’s simple pooled risk ratio of ~2.3 [11], likely because we formally account for study weights and include studies with no difference. A more recent systematic review by Hidajat et al. also reported a risk ratio of ~0.49 (buried versus exposed) for infection [10], almost identical to the results of the study by Chen et al. [7] and ours, reinforcing that multiple analyses of overlapping data have come to the same conclusion.

Importantly, several included studies, such as those by Koç et al. [4] and Terndrup et al. [9], showed no statistically significant difference between exposure methods. Incorporating these neutral findings ensures that the overall conclusion is not disproportionately influenced by the few positive studies.

However, the conclusion of the overall analysis shows that burying K-wires roughly halves the risk of a pin site infection.

Clinical Interpretation

The clinical significance of pin site infections in the hand/wrist should be kept in perspective. Most infections in these series were mild (Oppenheim grade I or II: redness or discharge treatable with local care and oral antibiotics) [4]. These patterns were consistent across studies, although some institutions routinely used theatre for buried wire removal regardless of complication status, reflecting protocol-based rather than complication-driven decision-making.

For instance, Ridley et al. found 52 (7.5%) patients managed with only oral antibiotics and pin retention [6]. Only six (0.9%) patients in their entire cohort required pin removal and IV antibiotics [6]. Similarly, Koç et al. noted that in both groups, most infections were grade I and resolved with a week of oral co-amoxiclav [4]. Severe infections were very infrequent, but they did occur exclusively or predominantly in the exposed wire groups in our review. For example, the only cases of osteomyelitis or deep abscess were in exposed wire patients (one case in the study by Koç et al. [4] and one case in the study by Ridley et al. [6] required surgical debridement). From a surgeon’s standpoint, burying wires might serve as a form of “insurance” against these worst-case infections. Plastic and orthopaedic surgeons who treat high volumes of hand fractures have anecdotally observed disastrous outcomes from neglected pin infections (e.g., flexor tendon sheath infection or joint sepsis [1]), which provides some rationale for the cautious approach of burying wires in certain cases despite the inconvenience.

Patient and Surgeon Preferences

Patients may have differing preferences: exposed wires avoid a second operation but require pin care vigilance, and buried wires eliminate external hardware but necessitate operative removal. The decision to bury or not often comes down to surgeon preference and patient-specific factors [12]. Our findings can inform that shared decision. For the surgeon, exposed wires are attractive due to quick application and removal, as reflected in a UK survey, where ease of removal was the top reason for not burying. This convenience also translates to efficiency in the clinic (no need for a second trip to the theatre). Orthopaedic surgeons in some settings may also prefer exposed wires if expecting to convert to another procedure or to assess reduction easily (although in fractures included here, conversion was rarely needed). On the other hand, surgeons who bury wires cite the reduced infection risk as the justification [12]. For the patient, having protruding wires can be frightening or uncomfortable; pins can snag on clothing or trigger anxiety about infection. Some patients, especially those who are non-local or have difficulty attending multiple visits, might prefer burying so that there is one less thing to manage during recovery (no pin care needed at home). However, burying obligates them to a minor surgery later, which many patients do not anticipate happily [4]. Our data suggest that for most straightforward hand fractures in healthy patients, leaving K-wires exposed is safe (infection risk is ~10% and usually minor, with essentially no impact on union) and spares the patient an extra procedure. This resonates with Wormald et al.’s paediatric elbow findings that non-buried wires did not increase infection or non-union and were more cost-effective [11]. On the other hand, for high-risk situations, for example, a metacarpal fracture in a young manual worker (higher infection tendency [4]) or a patient with factors such as diabetes or poor hygiene, a surgeon might lean towards burying wires to minimise any infection that could jeopardise the outcome. Indeed, some authors have suggested an individualised approach: bury wires in contamination-prone scenarios (open injuries and patients unlikely to keep pin sites clean) and leave them exposed otherwise [4].

Conditional Recommendations

Both orthopaedic and plastic hand surgeons can take away a few key points for clinical practice from this review.

Exposed K-wires are generally effective and low-risk for most hand and distal radius fractures. They eliminate the need for a second procedure and result in similar union rates as buried wires. Surgeons should ensure patients/caregivers are educated in pin site hygiene to keep infection rates at the low end (some centres have reported <5% infection with rigorous pin care protocols).

Buried K-wires do confer a lower incidence of pin site infection, particularly reducing superficial infections and almost eliminating the chance of a patient needing early hardware removal or developing a deep infection. This can be advantageous in fractures where even a brief infection-related delay in rehabilitation could be problematic (e.g., complex intra-articular fractures requiring absolute stability). Burying is also a consideration in patients who absolutely cannot afford an infection (e.g., immunocompromised patients), although none of the studies specifically addressed immunosuppressed or diabetic subgroups; interestingly, Ridley et al. found that diabetes and steroid use did not significantly affect infection rates in their cohort [6].

Metacarpal Fractures

Our analysis indicates they benefit most from burying in terms of infection reduction. A practical approach could be to bury K-wires for metacarpal shaft fractures (which often have more soft tissue movement and higher infection risk [4]), while leaving them exposed for phalangeal fractures, which inherently have lower infection risk and for which easy wire removal can facilitate earlier mobilisation of fingers.

Distal Radius Fractures

The evidence, albeit limited, favours burying wires if possible, as pin track infection rates in distal radius pinning can reach 20% when exposed [4]. The trial by Hargreaves et al. both recommended burying wires in the distal radius after noting high infection rates with percutaneous pins [1]. Many orthopaedic surgeons, however, treat distal radius fractures with alternative methods (plating or external fixators) if concerned about infection, so this decision might be case-dependent (e.g., bury if the patient has to have K-wires and has risk factors for infection).

Pin Site Care

An often under-discussed point is the role of pin site management. In these studies, exposed wire patients typically underwent weekly pin site dressing changes (saline or iodine gauze in Koç et al.’s protocol [4]). Good pin care can mitigate infection risk, and some centres even allow patients to shower or mobilise with exposed pins uncovered after initial healing, with no higher infection rates. Therefore, if choosing exposed wires, surgeons should implement a standardised pin care regimen. Conversely, burying wires avoids the need for pin care altogether, which in a noncompliant patient could be a reason to bury.

Study Limitations and Quality Considerations

The overall quality of evidence remains moderate. Only one true RCT has been done in adults (and one in children, although for a different joint [12]). Most data are retrospective. There is potential bias in that more difficult fractures might have been assigned to one technique. However, Ridley et al. helpfully report that surgeon preference, not fracture severity, dictated wire burial at their centre, and they controlled for fracture type, finding the burial benefit remained significant for metacarpals [6]. Another consideration is the per-wire versus per-patient issue. Some analyses (Hargreaves et al. [1] and Rafique et al. [3]) counted infections per wire, which could overestimate infection probability when multiple wires are in one patient. We used per-patient data where possible. The heterogeneity in our meta-analysis was low, suggesting consistency, but one must acknowledge the wide confidence intervals in smaller studies.

Future Directions

The persistent call is for a large, multicentre randomised trial to definitively answer this question [12]. Our meta-analysis suggests that to detect the ~5%-10% absolute difference in infection, a trial would need a few hundred patients per arm (as Ridley et al.’s power analysis also indicated [6]). Such an RCT (perhaps the proposed “WIRE” trial referenced by Wormald et al.’s group [11]) would ideally stratify by fracture type and could also incorporate patient-reported outcomes (pain, convenience and cosmetic satisfaction). Until then, this systematic review represents the best available evidence.

## Conclusions

Burying K-wires significantly reduces pin site infections, approximately halving the risk, and almost eliminates unplanned early removal, which may help maintain fracture stability. However, this benefit must be weighed against the need for a second operative procedure, increased healthcare resource use and added cost. Exposed K-wires, when paired with appropriate pin site care, produce acceptably low infection rates for most patients, avoid anaesthesia and do not compromise fracture union. The evidence indicates that both techniques are safe and effective, with the primary differences relating to infection risk and procedural burden. Thus, the decision between burying and exposing K-wires should be guided by fracture type, infection risk and the clinical context.

Burial may be particularly appropriate for higher-risk situations such as metacarpal fractures, patients with limited capacity to maintain pin hygiene, immunocompromised individuals or occupations where infection-related downtime has greater consequences. Conversely, leaving wires exposed may be preferable in low-risk patients, especially those with phalangeal fractures, where convenience, cost savings and avoidance of a second operation offer clear advantages. Patient preference should be incorporated into the decision, with appropriate education provided when wires are left exposed. Ultimately, a case-by-case strategy is the most appropriate approach, optimising patient outcomes while ensuring efficient use of healthcare resources. This balanced, individualised framework reflects current evidence and supports shared decision-making in hand and wrist fracture management.
